# Dimensional Information-Theoretic Measurement of Facial Emotion Expressions in Schizophrenia

**DOI:** 10.1155/2014/243907

**Published:** 2014-02-25

**Authors:** Jihun Hamm, Amy Pinkham, Ruben C. Gur, Ragini Verma, Christian G. Kohler

**Affiliations:** ^1^Department of Computer Science and Engineering, The Ohio State University, Columbus, OH 43210, USA; ^2^Southern Methodist University, Dallas, TX 75205, USA; ^3^Department of Psychiatry, Neuropsychiatry Section, University of Pennsylvania, Philadelphia, PA 19104, USA; ^4^Philadelphia Veterans Administration Medical Center, Philadelphia, PA 19104, USA; ^5^Department of Radiology, Section of Biomedical Image Analysis, University of Pennsylvania, Philadelphia, PA 19104, USA

## Abstract

Altered facial expressions of emotions are characteristic impairments in schizophrenia. Ratings of affect have traditionally been limited to clinical rating scales and facial muscle movement analysis, which require extensive training and have limitations based on methodology and ecological validity. To improve reliable assessment of dynamic facial expression changes, we have developed automated measurements of facial emotion expressions based on information-theoretic measures of expressivity of *ambiguity* and *distinctiveness* of facial expressions. These measures were examined in matched groups of persons with schizophrenia (*n* = 28) and healthy controls (*n* = 26) who underwent video acquisition to assess expressivity of basic emotions (happiness, sadness, anger, fear, and disgust) in evoked conditions. Persons with schizophrenia scored higher on *ambiguity*, the measure of conditional entropy within the expression of a single emotion, and they scored lower on *distinctiveness*, the measure of mutual information across expressions of different emotions. The automated measures compared favorably with observer-based ratings. This method can be applied for delineating dynamic emotional expressivity in healthy and clinical populations.

## 1. Introduction

The ability to communicate emotions through facial expressions is crucial for interpersonal engagement. Altered facial expressions of emotions are viewed as characteristic impairments in schizophrenia [1, 2] that already feature prominently during early illness [3, 4] and may precede the onset of psychosis [5]. Assessment of facial expressions has traditionally relied on observer-based rating scales. The Scale for the Assessment of Negative Symptoms (SANS [2]) and the Positive and Negative Syndrome Scale (PANSS [6]) are commonly used by clinicians to measure facial expressions during interview settings, including the ratings of affective flattening and inappropriate affect. The limited range of quantitative scores obtained during clinical interviews that preferentially focus on positive symptoms without standard prompts to elicit changes in expressivity decrease the ecological validity of facial expression ratings.

An alternative line of investigation of facial expressions of emotions has moved away from clinical assessment of expressions and examined local facial changes based on muscle movements or qualitative assessment of dynamic changes associated with facial expressions. The Facial Action Coding System (FACS [7, 8]) provides detailed descriptions of individual facial muscle movements in multiple facial regions. FACS and some of its adaptations have been applied to schizophrenia [9–13]. However, the FACS procedure requires extensive training and is time-consuming, which makes it difficult to perform in large-scale studies. Due to the prohibitive amount of time to rate videos, the FACS procedure is usually performed for static emotional displays. The Facial Expression Coding System (FACES [14]) can classify dynamic facial expressions with details such as valence, type, and intensity of emotions as determined by a rater. FACES ratings of videos of evoked emotions in schizophrenia have shown significant flatness and inappropriateness during spontaneous expressions [15, 16], as well as for simulated [17] and evoked expressions [18].

Media for capturing facial expressions have included still photographs [18–20], videotapes [9–13, 15, 17, 21, 22], and electromyographic recordings [23–25]. Videotaped acquisition offers the advantage of capturing duration and frequency of emotion expressions. However, analysis of such lengthy data sets has been limited to more global assessment of positive and negative emotion expressions, rather than changes in specific face regions. Common measurements of emotion expressions have included recognition rates of expressions [19, 21, 26–28] and FACS-derived measures without analysis of specific AUs [9–12, 15, 17]. Other methods have included computerized face morphometry [25, 27] and electromyographic measurements [23–25, 29, 30] that can measure minute muscle activations, albeit limited to select face regions, for example, zygomatic and corrugator muscles that are typically utilized in frowning and smiling expressions. An interesting and frequent finding arising from these investigations has been the possible disconnectedness between emotion expressions and subjective emotion experience [18, 30, 31], which in turn may affect social interactions in schizophrenia.

In an effort to derive more objective and scalable measurement of facial expressivity, we have pursued computerized measurement of facial expressions that were obtained during standardized procedures to elicit emotion expressions. Initial efforts based on manually defined face regions from still images in healthy actors [32] and persons with schizophrenia [33] showed significant group differences in expressions of happiness, sadness, anger, and fear. Wang et al. [34] refined the method to allow automated analysis of videos and demonstrated different frequencies of four emotions in three exemplar participants. To account for low intensity and ambiguous expressions of emotions, Hamm et al. [35] proposed automated measurement of individual facial muscles using FACS parameters and demonstrated the differences of facial muscle activities in four patients and controls. However, the implications of altered individual facial muscle movements on global expressivity and respective differences in schizophrenia, where facial expressions are commonly impaired, remain to be better elucidated. In this paper, we present novel measures of facial expressivity, based on individual muscle movements that are derived from information theory [36] and applied these measures to matched groups of schizophrenia and control subjects. Information theory originated in the 1950s from the field of applied mathematics to analyze the capacity of communication channels. The method has provided powerful tools to analyze human performance measures in psychological experiments [37] and a framework for understanding emotional communications [38]. Information theory has also been successfully used in biomedical investigations, such as processing capacity of neural coding [39], complexity in electrocardiogram sequences [40], and electroencephalography in Alzheimer's disease [41] and schizophrenia [42].

As an extension of our previous work that examined both observer-based and computerized measurements of facial expressivity of emotions, we tested sensitivity of the information-theoretic measures in characterizing and quantifying affective expression deficits in schizophrenia, and we examined their relationship with observer-based ratings of inappropriate and flattened emotion expressions. We applied two-dimensional measures of facial expressivity that can be computed objectively from videos of facial expressions without requiring observer-based ratings: (1) *ambiguity* of facial expressions within a single emotion and (2) *distinctiveness* of facial expressions across separate emotions. These measures correspond to the two most important information-theoretic quantities of (1) *conditional entropy* and (2) *mutual information*. Briefly, *ambiguity* is the amount of uncertainty in a person's facial muscle patterns during expression of a single emotion as contrasted with consistency of the pattern. A person whose facial muscle pattern is only brief or is variable during emotion expression will be less effective in communicating his or her intended emotion to another person. *Distinctiveness* is the capacity of a person to express different emotions succinctly through facial muscles. A person who is unable to produce distinct facial patterns for different emotions will also be less effective in communicating a specific emotion. We anticipated that *ambiguity* and *distinctiveness* measures can be applied to large data sets of dynamic expressions and they can capture aspects of expressivity deficits that would improve our understanding of emotion expression abilities in persons with schizophrenia. In addition, although representing different theoretical constructs, we examined whether information-theoretic measures correlate with observer-based ratings such as inappropriate and flattened affect.

## 2. Methods

### 2.1. Subjects

We collected videos of healthy controls and persons with schizophrenia for a neuropsychiatric study of emotions under an approved IRB protocol at the University of Pennsylvania. After describing the study to the subjects, we obtained written informed consents, including consent to publish pictures. There were 28 outpatients with a DSM-IV diagnosis of schizophrenia and 26 healthy controls balanced in gender, race, age, and parental education. All patients were clinically stable without hospitalization for at least 3 months prior to research assessment and had been maintained on their present medication for the past month. Presence of significant acute extrapyramidal symptoms as evidenced by a score of 1 or higher on at least 2 of the rigidity or tremor items (items 2, 3, 4, 5, and 9) was exclusionary. Likewise presence of significant tardive extrapyramidal symptoms as evidenced by a score of 2 or higher on items 1–4 (facial and oral movements) was exclusionary. All patients were treated with second-generation antipsychotics that were converted to dosages equivalent to olanzapine (OLZ); two patients were also treated with first-generation antipsychotics that were converted to dosages equivalent to chlorpromazine (CPZ). All medication dosages were stable for the past month prior to testing and no patient was treated with anticholinergic medications. Pertinent demographic and clinical information is summarized in Table 1.

### 2.2. Emotion Elicitation Procedure and Video Acquisition

To test emotion expression ability, we followed the emotion elicitation procedure previously described [43] and adapted it for use in schizophrenia [13]. Videos were obtained for neutral expressions and for five universal emotions (happiness, sadness, anger, fear, and disgust). Before recording, participants were asked to describe biographical emotional situations, when each emotion was experienced in mild, moderate, and high intensities, and these situations were summarized as vignettes. Subsequently, subjects were seated in a brightly lit room where recordings took place, and emotional vignettes were recounted to participants in a narrative manner using exact wording derived from the vignettes. The spontaneous facial expressions of the subjects were recorded as videos. Before and between the five emotion sessions, the subjects were asked to relax and return to a neutral state. The duration of each session was about two minutes (110 ± 54 sec).

### 2.3. Video Processing and Automated FACS

**Figure 1:**
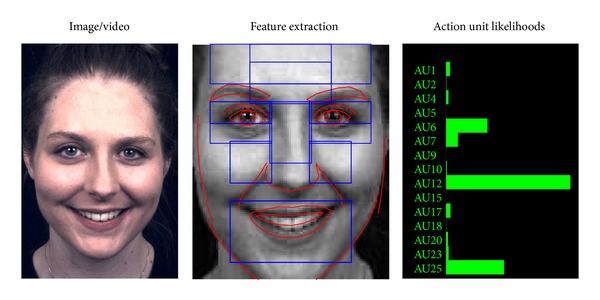
Automated Action Unit recognition system. For each frame of a video, geometric changes in facial components (red curves) are automatically tracked, and textural changes due to temporary wrinkles are detected in multiple regions of interest (blue boxes). These extracted features are fed through pattern classifiers to yield activity of each Action Unit (green bars: longer bar means higher activity).

Our group has developed an automated facial coding procedure that can score intensity of facial muscle activity known as Action Units (AUs [7, 8]) by computerized analysis of videos. The details of the system and the validation of accuracy appeared in Hamm et al. [35], and here we describe key components of the system and how we used it to measure information (see Figure 1). Videos acquired in evoked emotions were analyzed frame by frame. For each frame, geometric changes in facial components such as eyes, eyebrows, nasolabial line, and lips were automatically tracked, and textural changes due to temporary wrinkles were detected in multiple regions of the face. To increase the reliability of the tracking, a small fraction (~3%) of frames from videos in which the face leaves the screen (i.e., nonfrontal) were automatically discarded from analysis. Extracted geometric and texture features were then passed through pattern classifiers to yield intensity of the following 15 AUs: AU1 (Inner Brow Raiser), AU2 (Outer Brow Raiser), AU4 (Brow Lowerer), AU5 (Upper Lid Raiser), AU6 (Cheek Raise), AU7 (Lid Tightener), AU9 (Nose Wrinkler), AU10 (Upper Lip Raiser), AU12 (Lip Corner Puller), AU15 (Lip Corner Depressor), AU17 (Chin Raiser), AU18 (Lip Puckerer), AU20 (Lip Stretcher), AU23 (Lip Tightener) and AU25-27 (Lips Part and Mouth Open). The other AUs than these 15 AUs were not used since they were too infrequently in the recorded videos.

### 2.4. Computation of *Ambiguity* and *Distinctiveness* of Facial Expressions

From the distribution of 15-dimensional continuous variables collected from videos, we computed information-theoretic measures of expressivity for each subject. Specifically, we measure *ambiguity* of facial expression patterns within each emotion and *distinctiveness* of facial expression patterns across emotions. These two measures correspond to conditional entropy and mutual information, which are the fundamental variables of information theory [36]. Interpretations of the two information-theoretic measures depend on the experiments being conducted, and *ambiguity* and *distinctiveness* are specific interpretations of our experimental results with the spontaneous expression of emotions. In psychology, the two measures have been called equivocation and information transmission, respectively, in the context of absolute judgment tasks [44].

Computation of *ambiguity* and *distinctiveness* requires estimation of differential entropy from facial muscles. Differential entropy is the amount of uncertainty in continuous probabilistic distributions from which we derive mutual information and conditional entropy. Let *x* denote the (discrete) emotional state of an individual and *y* denote the (continuous and multivariate) facial muscle activity. Differential entropy is then defined by *H*(*p*(*y*)) = *E*[−log⁡*p*(*y*)] = −∫_ℝ^*d*^_ *p*(*y*)log⁡*p*(*y*)*dy*. Unlike discrete entropy, differential entropy is a relative measure and can have negative values; a univariate Gaussian distribution with *σ* = (2*πe*)^−1/2^ has zero differential entropy, and Gaussians with narrower or wider peaks have negative or positive entropy, respectively. Since we do not know *p*(*y*)  *a priori*, the differential entropy has to be estimated from samples, much like estimation of mean and variance. Several estimation methods have been proposed, including adaptive partitioning, kernel density estimation, and nearest neighbor estimation (see Beirlant et al. [45] for a review). We used a *k*-nearest neighbor estimator of entropy [46–48].

*H*(*p*(*y*))≈(*d*/*n*)∑_*i*_^*n*^log⁡*ρ* + *v*_*d*_ − *ψ*(*k*) + log⁡⁡*n*. The *v*_*d*_ = *π* ^*d*/2^/Γ(*d*/2 + 1) is the volume of a *d*-dimensional unit sphere, where Γ is the gamma function Γ(*z*) = ∫_0_^*∞*^ *t* ^*z*−1^ *e* ^−*t*^ *dt* and *ψ*(*z*) is the digamma function *ψ*(*z*) = Γ(*z*)′/Γ(*z*). The only free parameter of nearest-neighbor estimate is the size of the neighbors *k* for which we used the heuristic rule [47].

*k = round *(*n* ^1/2^ + 0.5), where *n* is the number of samples. For numerical stability, we also added negligible amount ( = 10^−8^) of random noise to the data while computing entropy.

In our experiments, the conditional entropy is defined as *H*(*y* | *x*) = ∑_*x*=*x*_*i*__ *p*(*x*_*i*_)*H*(*y* | *x*_*i*_) = ∑_*x*=*x*_*i*__ *p*(*x*_*i*_)∫−*p*(*y* | *x*_*i*_)log⁡(*p*(*y* | *x*_*i*_))*dy*, which is the average entropy of facial expression per emotion computed from facial muscle activity *y* in the video of each emotion *x* with equal priors for each emotion (*p*(*x*) = 1/5). We refer to this conditional entropy as *ambiguity* in the following context: when an individual's facial expression is consistent within each emotion, the conditional entropy is low, and when the expression is varying and ambiguous within each emotion, the conditional entropy is high.

The mutual information *I*(*x*, *y*) can be computed from *I*(*x*; *y*) = *H*(*y*) − *H*(*y* | *x*). Mutual information between discrete and continuous variables, as in our case, is also known as Jensen-Shannon divergence [49, 50] and is nonnegative and bounded. By reformulating *I*(*x*; *y*) as the average KL-divergence [51] between conditional *p*(*y* | *x*) and marginal *p*(*y*) distributions *I*(*x*; *y*) = *KL*(*p*(*x*, *y*)||*p*(*x*)*p*(*y*)) = ∑_*x*_ *p*(*x*)∫*p*(*y* | *x*)log⁡⁡(*p*(*yx*)/*p*(*y*))*dy* = ∑_*x*_ *p*(*x*)*KL*(*p*(*y* | *x*)||*p*(*y*)), we notice that mutual information measures the average distance of emotion-specific facial expression pattern (*p*(*x* | *y*)) from the patterns of all emotions combined (*p*(*x*)). Hence our choice of the term *distinctiveness* is for mutual information.

### 2.5. Interpretation of *Ambiguity* and *Distinctiveness *Measures

In this paper, we report the *ambiguity* and the *distinctiveness* as *z*-scores ( = (*x* − *m*)/*s*) instead of their raw values for an easier interpretation, where the mean and the standard deviation are computed using all subjects and conditions. We do this because these values are unitless and dependent on the experimental setting and therefore meaningful only in a relative sense. For example, if we use six basic emotions including “surprise” instead of five, the absolute values of *ambiguity* and *distinctiveness* will change. However, the difference of values in diagnostic groups or conditions still measures the relative amount of the *ambiguity* and *distinctiveness* of facial expressions and provides meaningful information. Note that the standardization of raw values using *z*-scores does not affect the subsequent statistical analysis.

Lastly, the *ambiguity* and the *distinctiveness* are computed across all emotions and not for individual emotions. While it is possible to analyze *ambiguity* for each emotion, pooling the values across emotions results in more reliable measures.

### 2.6. Observer-Based Measures

*Validation of Ambiguity and Distinctiveness.* To verify that the information-theoretic measures agree with an observer's interpretation of *ambiguity* and *distinctiveness* from videos, the following criteria for manual scores from 0 to 4 were defined, and each video was rated by an observer blind to the diagnosis of subjects. For *ambiguity*, which was rated for videos of each emotion, 0 meant very consistent (with only single facial expression pattern in the single video of an emotion) and 4 meant very ambiguous (with more than four different facial expression patterns in the video of an emotion). Scores 1 to 3 corresponded to intermediate levels of *ambiguity* (with 1, 2, and 3 major facial expression patterns in a video, resp.). For *distinctiveness*, which was rated across videos of five emotions of a single subject, 0 meant the five emotional videos were totally indistinguishable in representing the target emotions, and 4 meant all five videos were distinctive and representative of the target emotions. Scores 1 to 3 corresponded to intermediate levels of *distinctiveness* (with 1, 2, and 3 videos of distinctive emotions, resp.).

*Observer-Based Ratings of Facial Expressions*. To compare information measures with previous observer-based ratings, flatness and inappropriateness of facial expression from the Scale for the Assessment of Negative Symptoms (SANS) were adapted to video-based ratings [33]. Two raters scored each video with separate scores for flat and inappropriate affect, ranging from 0 (none) to 4 (extremely flat or inappropriate). SANS raters knew the intended emotion of the video but not the diagnosis of subjects. Video-based SANS was based on a 5-point rating, similar to observer-based SANS. Ratings that differed by 2 or more points were reviewed for consensus and final ratings were averaged.

### 2.7. Statistical Analysis

The following data analysis was performed to demonstrate applicability and validity of the measures of *ambiguity* and *distinctiveness. *Group-based comparisons for *ambiguity *and* distinctiveness* of expressions involved were carried out via two-way ANOVA of *ambiguity* and *distinctiveness* separately, using sex and diagnosis as grouping factors. We also measured the effect size of diagnosis by Cohen's *d *( = (*m*_1_ − *m*_2_)/(0.5(*s*_1_^2^ + *s*_2_^2^))^1/2^). Validation of computerized measures of *ambiguity* and *distinctiveness* against observer-based measures was performed by Pearson correlations, where higher values meant better agreement between the computerized and observer-based measures. For observer-based ratings of inappropriateness and flatness of expressions we performed separate two-way ANOVA using sex and diagnosis as grouping factors. We also measured the effect size of diagnosis by Cohen's *d*. For the purposes of interpretability and comparison with alternative measures of symptom severity, we performed multiple regression analysis to study the explanatory power of the computerized measures of *ambiguity* and *distinctiveness* for observer-based ratings of inappropriate and flat affects, in which computerized measures were used as independent variables and each of the observer-based ratings was used as a dependent variable. We performed multiple regression also in the other direction, in which observer-based ratings were used as independent variables and each of the computerized measures was used as a dependent variable.

## 3. Results

### 3.1. *Ambiguity* and *Distinctiveness* of Emotion Expressions

*Ambiguity* of expression, averaged across emotions, showed a strong effect of diagnosis (*F* = 12, *df* = 1,53, *P* = 0.0012), but no effect of sex (*F* < 0.001, *df* = 1,53, *P* = 0.99) nor interaction (*F* = 0.78, *df* = 1,53, *P* = 0.38) by two-way ANOVA. Patients showed higher *ambiguity* (0.41 ± 1.0) than controls (−0.44 ± 0.79), with a large effect size of 0.93 (Cohen's *d*). Likewise, *distinctiveness* of expression showed a strong effect of diagnosis (*F* = 8.3, *df* = 1,53, *P* = 0.0057), but no effect of sex (*F* = 0.056, *df* = 1,53, *P* = 0.81) nor interaction (*F* = 0.26, *df* = 1,53, *P* = 0.61) by two-way ANOVA. Patients showed lower *distinctiveness *(−0.37 ± 0.88) than controls (0.40 ± 0.98) with a large effect size of Cohen's *d* = 0.83.

**Figure 2:**
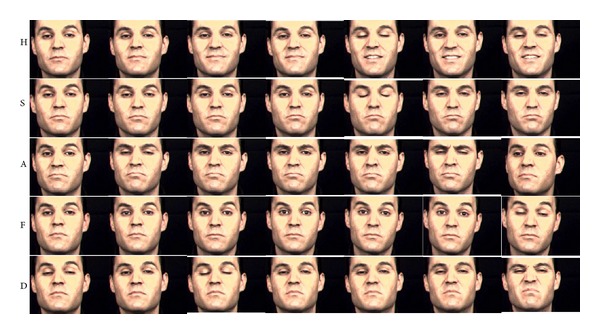
Sample videos of a subject with low *ambiguity* and high *distinctiveness*. H, S, A, F, and D stand for happiness, sadness, anger, fear, and disgust. Each row shows sample images evenly chosen from a video of the emotion. Facial expressions of low *ambiguity* and high *distinctiveness* are consistent within each emotion and also different across emotions, which makes each emotion well recognizable.

**Figure 3:**
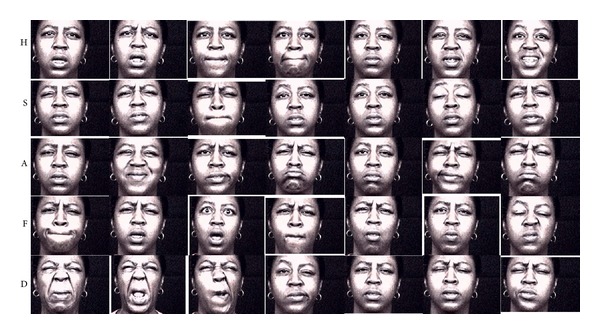
Sample videos of a subject with high *ambiguity* and low *distinctiveness*. High *ambiguity* and low *distinctiveness* appear as inappropriate facial expressions, due to the lack of consistent patterns within each emotion and the overlap of patterns across emotions.

**Figure 4:**
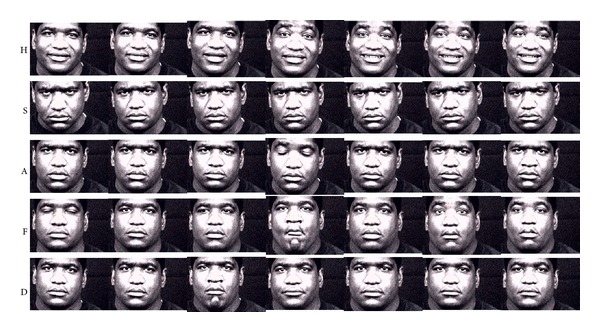
Sample videos of a subject with high *ambiguity* and high *distinctiveness*. High *ambiguity* and high *distinctiveness* also appear as inappropriate facial expressions due to the lack of consistent patterns within each emotion.

**Figure 5:**
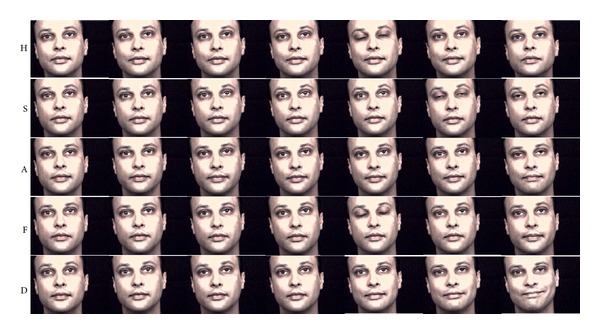
Sample videos of a subject with low *ambiguity* and low *distinctiveness*. Facial expressions of low *ambiguity* and low *distinctiveness* present themselves as muted or flat expressions throughout all emotions.

The characteristics of *ambiguity* and *distinctiveness* measures are demonstrated with sample videos of subjects in Figures 2–5 that illustrate the expressions of subjects with ratings for low *ambiguity*/high *distinctiveness*, in contrast to subjects with ratings for low *ambiguity*/low *distinctiveness*, high *ambiguity*/low *distinctiveness*, and high *ambiguity*/high *distinctiveness*.

### 3.2. Observer-Basted Measures

*Validation of Ambiguity and Distinctiveness.* The computerized measures of *ambiguity* and *distinctiveness* were well correlated with the observer's scores of *ambiguity *(*r* = 0.71, *P* < 0.01) and *distinctiveness *(*r* = 0.60, *P* < 0.01). These correlations supported the notion of agreement between the computerized measures from information theory and the observer-rated measures from visual examination of the videos.

*Observer-Based Ratings of Facial Expressions*. For video-based expert SANS ratings, flatness of expression showed a moderate effect of diagnosis (*F* = 4.6, *df* = 1,53, *P* = 0.038), but no effect of sex (*F* = 3.9, *df* = 1, 53, *P* = 0.055) nor interaction (*F* = 0.19, *df* = 1,53, *P* = 0.66) by two-way ANOVA. Patients were more flat (2.0 ± 0.93) than controls (1.5 ± 0.91), with a medium effect size of 0.57. The inappropriateness of expression showed a strong effect of diagnosis (*F* = 9.6, *df* = 1,53, *P* = 0.0033), but no effect of sex (*F* = 0.99, *df* = 1,53, *P* = 0.33) nor interaction (*F* = 0.0047, *df* = 1,53, *P* = 0.95) by two-way ANOVA. Patients were rated higher on inappropriate affect (1.1 ± 0.65) than controls (0.63 ± 0.37)with a large effect size of 0.92.

### 3.3. Relationship between Computerized and Observer-Based Measures

We examined the relationships between information-theoretic measures of *ambiguity* and *distinctiveness* and observer-based measures of inappropriate and flattened facial expressions of emotions. Regression of *ambiguity* on observer-based measures was moderately predictive (*R* ^2^ = 0.13, *F* = 3.4, *P* = 0.040), and the coefficients from flatness and inappropriateness were −0.14 (*P* = 0.30) and 0.50 (*P* = 0.032), respectively. Regression of *distinctiveness* on observer-based measures was highly predictive (*R* ^2^ = 0.19, *F* = 5.8, *P* = 0.0056), and the coefficients from flatness and inappropriateness were −0.32 (*P* = 0.027) and −0.65 (*P* = 0.0063), respectively. Regression of flatness on computerized measures was moderately predictive (*R* ^2^ = 0.15, *F* = 4.3, *P* = 0.019), and the coefficients from *ambiguity* and *distinctiveness* were −0.33 (*P* = 0.025) and −0.34 (*P* = 0.013) respectively. Lastly, regression of inappropriateness on computerized measures was moderately predictive (*R* ^2^ = 0.15, *F* = 4.3, *P* = 0.018), and the coefficients from *ambiguity* and *distinctiveness* were 0.14 (*P* = 0.11) and −0.14 (*P* = 0.11), respectively.

## 4. Discussion and Conclusions

Impaired abilities of facial expression of emotions are common dysfunctions in schizophrenia that are associated with worse quality of life and poorer outcome [52, 53]. Clinical assessment of emotion expression abilities is typically obtained using observer-based rating scales administered during interviews that are not standardized to elicit emotion expressions and with limited potential to compare data across different studies and populations. More advanced observer-based measurements of facial expressions have been challenged by the complexity inherent in rating regional and global changes in dynamic facial expressions and their applications have been mainly limited to research. Nevertheless these investigations using standardized observer based ratings and electromyography have underscored that persons with schizophrenia exhibit altered expressions of volitional and of more fleeting spontaneous facial emotions that do not necessarily reflect their internal emotional state. Another challenge of measuring emotion expressions has been the importance to obtain emotion expressions that are genuine and naturalistic and not obscured by the artifice of the testing setting and methodology or influenced by rater fatigue. This is where automated computerized measurement offers an advantage over other methods to investigate dynamic expressions.

Our group has developed computerized assessment of emotion expression abilities that allows for objective measurement of facial expressions that are obtained in a standardized setting [32–35]. As an extension to these efforts, we have applied computerized assessments of facial expressivity to patients with schizophrenia and healthy controls to examine whether the novel methodology can elucidate differences in facial expression of emotions. Computerized information-theoretic measures focus on differences in *ambiguity*, which represents the measure of variability within the expression of a single emotion, and *distinctiveness*, which represents the measure of how distinctive facial expressions are for a particular emotion in comparison to other emotions. The computerized approach was validated by observer-based ratings of *ambiguity* and *distinctiveness*. In addition when computerized measures were predicted by observer-based ratings, *ambiguity* was positively related to inappropriateness, and *distinctiveness* was negatively related to both flatness and inappropriateness. Conversely, when observer-based ratings were predicted by computerized measures, flatness was negatively related to both *ambiguity* and *distinctiveness*, while inappropriateness was not significantly related with either measure alone, although it was significantly predicted by overall *ambiguity* and *distinctiveness*. As illustrated in Figures 2–5, computerized measures of *ambiguity* and *distinctiveness* were associated with facial expressions that, in the combination of low *ambiguity*/high *distinctiveness *(Figure 2), were well recognizable within each emotion and also different across emotions. High *ambiguity* with either low (Figure 3) or high (Figure 4) *distinctiveness* values appeared as inappropriate expressions that were either similar or different across emotions. The computerized measures also indicate flatness, as when facial expressions are not variable within an emotion (low *ambiguity*) and also indistinguishable across emotions (low *distinctiveness*) (Figure 5). These results suggest that the information-theoretic measures of emotion expressions are related to the observer-based ratings and computerized measures may provide quantifiable information on different causes of observed inappropriate affect. Specifically, inappropriateness of expression may result either from ambiguous and inconsistent facial expressions in each emotion regardless of *distinctiveness* across emotions. Flat affect, on the other hand, was mainly related to emotion expressions being indistinct across emotions.

Our computerized method employing information-theoretic measures offers several methodological advantages over currently available observer-based rating scales and FACS-based rating instruments, for example, our method being more objective and repeatable for any number of subjects, at the same time being less labor intensive and time-consuming. It is more objective than FACES since the method does not involve observer-judgment of emotions expressed and is less labor/time intensive than FACS and FACES since it is fully automated similar to EMG, without the inconvenience of physically placing electrodes on the participants' faces.

While we demonstrated the feasibility and applicability of these measures with schizophrenia patients and healthy controls, we are applying the analysis to a larger sample that can better reflect the range of individual differences in the respective populations. A major limitation of our approach is based on the fact that no computerized determination was made regarding emotional valence and such qualitative information could have enhanced the results based on information-theoretic measures of expressivity. Another limitation of the current study is that the induction procedure within the laboratory, while standardized, may lack ecological validity. A more naturalistic setting would include filming participants while they recount their own experiences. Unfortunately at the present state of this methodology, facial movements on account of speech would generate too much noise for our algorithms to overcome.

In conclusion, our method offers an automated means for quantifying individual differences in emotional expressivity, similar to what has been accomplished in the area of emotion recognition. This capability opens new venues for delineating emotional expressivity in healthy people and across clinical populations. As for investigations in healthy population, the automated procedure is well suited to examine both age and gender related changes in emotion expression abilities. Potential clinical applications may include repeated monitoring of facial expressions to investigate effects of disease progression and more general treatment effects or to measure targeted efforts to remediate emotion expression abilities. Automated comparisons need not be limited to select clinical populations and may allow for effective comparisons of emotion expressivity across different psychiatric disorders.

## Figures and Tables

**Table 1:** Demographic and clinical characteristics.

	Patient group	Control group
Gender (M : F)	14 : 14	16 : 10
Ethnicity		
(Caucasian : African-American : other)	9 : 16 : 3	8 : 15 : 3
Age		
Mean	32.3 ± 9.0	33.0 ± 8.6
Range	20–47 yrs	20–48 yrs
Duration of illness	13.00 ± 8.3	N/A
Range	2–33	N/A
Symptoms		
Positive (SAPS-total)	6.00 ± 8.5	N/A
Negative (SANS-total)	13.00 ± 8.3	N/A
Antipsychotics		
Chlorpromazine-equivalents (*n* = 28)	225 mg ± 247	N/A
Olanzapine-equivalents (*n* = 2)	13.6 mg ± 7.6	N/A
